# Co-Expression of TWIK-Related Acid-Sensitive K^+^ Channel 1 (TASK-1/KCNK3) and Platelet-Derived Growth Factor Receptor Alpha (PDGFRα/Pdgfra) in Adult Mouse Ovary

**DOI:** 10.3390/biomedicines13081941

**Published:** 2025-08-08

**Authors:** Byeonggyu Ahn, Eun-Jin Kim, Jin-Mok Kim, Sohi Kang, Sumi Hwang, Chang-Woon Kim, In Ae Cho, Jeong Kyu Shin, Eun-A Ko, Dong-Kun Lee, Dawon Kang

**Affiliations:** 1 Department of Physiology, College of Medicine and Institute of Medical Sciences, Gyeongsang National University, Jinju 52727, Republic of Korea; dksqudrb1184@naver.com (B.A.); eunjin1981@hanmail.net (E.-J.K.); 2 Department of Convergence Medical Science, Gyeongsang National University, Jinju 52727, Republic of Korea; anakang@gnu.ac.kr; 3 Department of Clinical Laboratory Science, Masan University, Changwon 2640, Republic of Korea; jmkim@masan.ac.kr; 4 Department of Anatomy, College of Medicine, Institute of Medical Sciences, Gyeongsang National University, Jinju 52727, Republic of Korea; 5 Department of Biomedical Laboratory Science, College of Health and Medical Science, Sangji University, Wonju 26339, Republic of Korea; hwang722@sangji.ac.kr; 6 Department of Obstetrics and Gynecology, Samsung Changwon Hospital, Sungkyunkwan University School of Medicine, Changwon 51353, Republic of Korea; kcwoon@naver.com; 7 Department of Obstetrics and Gynecology, College of Medicine, Gyeongsang National University, Jinju 52727, Republic of Korea; obgychoia@gnu.ac.kr (I.A.C.); 2848049@hanmail.net (J.K.S.); 8 Department of Physiology, College of Medicine, Jeju National University, Jeju 63243, Republic of Korea; koeuna@jejunu.ac.kr

**Keywords:** ovary, platelet-derived growth factor receptor alpha, potassium channel subfamily K member 3, theca cells

## Abstract

**Background/Objectives**: Platelet-derived growth factor receptor alpha (PDGFRα) is a receptor involved in cell growth and differentiation, with unclear roles in ovarian tissues and potential interactions with KCNK3 (potassium two-pore domain channel subfamily K member 3), a member of the two-pore domain K^+^ channel involved in cellular homeostasis. This study aims to map PDGFRα expression across mouse tissues and to explore its co-expression with KCNK3 in the ovary. **Methods**: We visualized PDGFRα expression using RNA-seq data from the genotype-tissue expression (GTEx) BodyMAP across 54 human tissues and Cap Analysis of Gene Expression (CAGE) data for various mouse tissues. In PDGFRα^EGFP^ mice expressing EGFP in PDGFRα^+^ cells, histological and fluorescence imaging were used to assess ovarian expression. Immunohistochemistry determined the co-localization of PDGFRα and KCNK3, and qPCR quantified their mRNA levels in the ovary, oviduct, and uterus. **Results**: PDGFRα showed high expression in human and mouse female reproductive tissues, particularly the ovary. In the PDGFRα^EGFP^ mouse model, PDGFRα was primarily found in the thecal layer and stromal cells, not in granulosa cells or oocytes. Immunohistochemistry indicated that 90.2 ± 8.7% of PDGFRα^+^ cells expressed KCNK3 in the ovarian stroma. qPCR revealed lower PDGFRα and KCNK3 expression in the ovary compared to the oviduct and uterus. **Conclusions**: This study shows that PDGFRα is predominantly expressed in ovarian stromal and theca cells and is highly co-localized with KCNK3, suggesting a potential role for PDGFRα^+^ cells in ionic regulation and their possible involvement in follicular development and ovarian physiology.

## 1. Introduction

Platelet-derived growth factors (PDGFs) are dimeric proteins functioning as potent mitogens and angiogenic factors. They can form homodimers (AA, BB, CC, DD) or heterodimers (AB), each with specific receptor affinities. PDGF-AA, PDGF-AB, and PDGF-BB activate PDGF receptor alpha (PDGFRα), while PDGF-BB and PDGF-DD preferentially bind to PDGFRβ, initiating downstream signaling through tyrosine kinase receptors [1,2,3,4,5]. These signaling events promote cellular functions, including proliferation, migration, and vascular remodeling [6,7]. The Human Protein Atlas reports that the PDGFRα mRNA is highly expressed in the human ovary, with corresponding protein levels most prominently detected in the ovary and endometrium (HPA, GTEx, and FANTOM5 datasets, cited in references). Consistently, mouse transcriptomic data from the FANTOM5 project show strong PDGFRα expression in the uterus and ovary. PDGFRβ also exhibits substantial ovarian expression, although it is slightly lower than that of PDGFRα.

Earlier studies have identified PDGF ligands and receptors in oocytes within primordial, primary, and early-developing follicles, contributing to the maturation of primordial follicles [8,9]. PDGFs and other growth agents have been recognized as critical modulators in transitioning from primordial to primary follicles, with variations in mRNA expression levels supporting their role [10]. PDGFs act as angiogenic factors, recruiting smooth muscle cells and pericytes to stabilize and reinforce blood vessels, a process crucial for ovarian function [9,11,12]. Angiogenesis in the ovary is essential for supplying nutrients and hormones that support follicular growth and corpus luteum development [13,14,15]. Consequently, disruptions in PDGF signaling may result in menstrual irregularities, amenorrhea, and infertility and potentially contribute to ovarian cancer development [16,17]. Growing evidence underscores the essential role of PDGF signaling and expression in regulating ovarian function across diverse species.

Two-pore domain potassium (K_2P_) channels are crucial in establishing the resting membrane potential and regulating cellular excitability across various tissues. These channels are fundamental to physiological processes such as hormone secretion, smooth muscle tone, and sensory transduction [18,19,20,21]. Among the K_2P_ channel family, the TWIK-related acid-sensitive K^+^ (TASK) channels are notably expressed in the female reproductive tract, including the ovary, uterus, and placenta [22,23,24,25]. This widespread distribution suggests that K_2P_ channels, particularly the TASK subfamily, may participate in diverse aspects of reproductive physiology. TASK-1, officially known as potassium two-pore domain channel subfamily K member 3 (KCNK3), is a member of the K_2P_ channel family and is prominently expressed in PDGFRα^+^ cells in mice [26]. KCNK3 channels are regulated by physiological cues such as oxygen tension, pH, and hormonal signals [27,28]. KCNK3 is localized in Leydig and peritubular cells in the testis, expressing PDGFRα [29]. These findings in male reproductive tissues raise the possibility of a conserved interaction between PDGFRα and KCNK3 across sex-specific organs. Notably, theca cells in the ovary are interstitial cells that are functionally analogous to Leydig cells, sharing a steroidogenic role within their respective gonadal environments [30,31].

Based on this parallel, we hypothesized that PDGFRα and KCNK3 may be co-expressed in the ovarian theca or stromal compartments. To test this, we examined their expression patterns using PDGFRα^EGFP^ reporter mice, which express enhanced green fluorescent protein under the control of the endogenous PDGFRα promoter, enabling the precise visualization of PDGFRα-positive cells in ovarian tissue.

## 2. Materials and Methods

### 2.1. Transcriptomic Data Analysis of PDGFRα Expression

To analyze the tissue-specific expression of PDGFRα across various human tissues, Human BodyMap RNA-seq data were obtained from the Genotype-Tissue Expression (GTEx) project. The dataset was accessed via the GTEx Portal (https://gtexportal.org/home/gene/PDGFRA) and the database of Genotypes and Phenotypes (dbGaP; accession number phs000424.v8.p2) on 24 July 2024. This analysis included transcriptomic data from 54 non-diseased tissues, representing nearly 1000 individuals. Differential expression analysis focused explicitly on PDGFRα in various female reproductive tissues. For comparative analysis in mice, transcriptomic data were sourced from the Mouse Genome Informatics (MGI) database. Functional Annotation of the Mammalian Genome 5 (FANTOM5) project Cap Analysis of Gene Expression (CAGE) was used to assess PDGFRα expression across various female tissues in mice, providing insight into the tissue-specific expression patterns. FANTOM5 CAGE data were downloaded from https://fantom.gsc.riken.jp/5/datafiles/reprocessed/mm10_latest/extra/CAGE_peaks_expression/ on 24 July 2024.

### 2.2. Animal Models and Housing Conditions

This model was previously utilized in our study to investigate the expression pattern of PDGFRα in the mouse testis, which provides a foundational basis for the current experiments [29]. Although PDGFRα is known to be highly expressed in the human ovary based on transcriptomic datasets, its localization in mouse ovarian tissue has not been established. Therefore, we used the PDGFRα^EGFP^ model to explore and visualize the spatial expression of PDGFRα^+^ cells in the mouse ovary. For this study, heterozygous PDGFRα^EGFP^ male mice (B6.129S4-Pdgfra^tm11 (EGFP)Sor^/J, Stock No. 007669, aged 6–7 weeks) were mated with wild-type (WT) females of the same age, both procured from Jackson Laboratory (Bar Harbor, ME, USA). The PDGFRα^EGFP^ mouse line carries an H2B-EGFP fusion gene under the control of the native *Pdgfra* promoter, resulting in fluorescence that mirrors the endogenous expression pattern of PDGFRα. Since homozygous expression of this gene leads to embryonic lethality, breeding was carried out using heterozygous PDGFRα^EGFP^ males mated with WT females to ensure the production of viable offspring for genotyping and experimental purposes. For the experiments described in this study, eight 12-week-old heterozygous PDGFRα^EGFP^ females were utilized. All mice were housed under specific pathogen-free conditions with a controlled 12 h light–dark cycle at a temperature range of 20–24 °C and a humidity level of 50–60%. The mice had ad libitum access to food and sterile water. All animal experiments were performed according to ethical guidelines approved by the Gyeongsang National University Animal Care and Use Committee under protocol number (GNU-240527-M0112).

### 2.3. Hematoxylin and Eosin (H&E) Staining

Histological examination of ovarian tissues was conducted using H&E staining, following the protocol outlined in a previous study [32]. Ovarian tissues were fixed overnight at 4 °C in 4% paraformaldehyde and then washed with 0.1 M PBS. The tissues were subsequently embedded in paraffin and sectioned into 5 μm thick slices. The paraffin sections were air-dried on gelatin-coated slides, deparaffinized, and rinsed with tap water. The sections were stained with hematoxylin for 5 min, followed by staining with eosin for another 5 min. A graded series of ethanol (70–100%, each for 3 min) was used for dehydration, and sections were cleared with xylene. The slides were mounted using a Permount mounting medium (Fisher Chemical, Geel, Belgium). This standard H&E staining protocol has been previously optimized and validated for ovarian tissue to ensure morphological clarity and consistency [33]. The stained sections were examined and photographed using an Olympus BX61VS microscope (Olympus, Tokyo, Japan). To ensure consistency, five different sections from each sample were analyzed.

### 2.4. Immunohistochemistry (IHC)

Following deparaffinization, tissue sections were permeabilized with 0.1% Triton X-100 in PBS for 10 min at room temperature. After three washes with 1× PBS, sections were incubated for 60 min at room temperature in a blocking solution containing 1.5% bovine serum albumin (BSA) in 1× PBS. For KCNK3 detection, sections were incubated overnight at 4 °C with an Alexa Fluor^®^ 405-conjugated anti-KCNK3 monoclonal antibody (1:200; Novus Biologicals, Centennial, CO, USA). This directly conjugated primary antibody was selected to minimize background staining and avoid non-specific binding frequently observed with secondary antibody-based detection in preliminary tests. After incubation, tissues were washed three times with cold 1× PBS and mounted using Gel/Mount™ (Biomeda Corp., Foster City, CA, USA). Images were captured using an Olympus FV1000 confocal laser scanning microscope (Olympus, Tokyo, Japan). To assess co-localization of 17α-hydroxylase (CYP17A1) and PDGFRα, permeabilized ovarian sections were incubated overnight at 4 °C with a mouse monoclonal anti-CYP17A1 antibody (1:200; Santa Cruz Biotechnology, Dallas, TX, USA; sc-374244). After three 5 min PBS washes, a fluorophore-conjugated secondary antibody (anti-mouse TEXAS Red, 1:500; Thermo Fisher Scientific, Carlsbad, CA, USA) was applied for 2 h at room temperature. Sections were then rewashed in PBS (3 × 5 min), mounted with Gel/Mount™ (Biomeda Corp.), and visualized using confocal microscopy. In this study, nuclear counterstaining was not performed. The PDGFRα^EGFP^ mouse line expresses EGFP fused to histone H2B, resulting in nuclear-localized fluorescence. Applying a DNA-binding dye such as DAPI could interfere with or obscure the EGFP signal due to spectral overlap, potentially compromising the accuracy of PDGFRα detection. Therefore, nuclear counterstaining was deliberately omitted to preserve the specificity and clarity of EGFP-based nuclear fluorescence. Although a classical positive control using overexpressed proteins in cell lines was omitted, we employed robust antibody validation strategies. For KCNK3, a directly conjugated primary antibody (Alexa Fluor^®^ 405) was used to minimize non-specific binding and reduce background fluorescence. For CYP17A1, an established marker of theca cells, endogenous expression was leveraged as an internal control, and the staining pattern was consistent with its known thecal localization. Negative controls with antibody omission (NC) were also included to verify signal specificity.

For quantification of co-localization, IHC images were analyzed using ImageJ2 software (NIH, Bethesda, MD, USA). Five non-overlapping fields from each ovarian section (n = 4 ovaries) were randomly selected. The number of PDGFRα^+^ cells and cells co-expressing PDGFRα and KCNK3 were manually quantified using the Cell Counter plugin in ImageJ2. Fluorescence signals were classified as positive only if their intensity was distinguishable from the background fluorescence established in NC sections, where the primary antibody was omitted. Signals that matched or fell below the background intensity were excluded from analysis. This threshold-based approach was consistently applied across all samples to ensure objective and reproducible identification of marker-positive cells. The percentage of KCNK3^+^/PDGFRα^+^ cells was determined by dividing the number of double-positive cells by the total number of PDGFRα^+^ cells and multiplying by 100. Data were expressed as mean ± standard deviation (S.D.).

### 2.5. RNA Isolation and Quantitative Real-Time PCR

Mouse tissues were immediately frozen in liquid nitrogen upon collection and stored in a deep freezer until further use. For RNA extraction, the frozen tissues were homogenized in TRIzol reagent (Invitrogen, Thermo Fisher Scientific, Carlsbad, CA, USA), and total RNA was isolated following the manufacturer’s instructions. The isolated RNA was then reverse transcribed into complementary DNA (cDNA) using the RevertAid First Strand cDNA Synthesis Kit (Invitrogen), with oligo (dT) (18-mer) primers. The synthesized cDNA was used as a template for quantitative real-time PCR (qRT-PCR), performed with the amfiSure qGreen Q-PCR Master Mix (2X) (GenDEPOT, Altair, TX, USA). The specific primers used for the reactions are listed in Table 1. All qPCR reactions were performed on a QIAquant 96 2plex system (Qiagen, Hilden, Germany). PCR amplification was performed in a 10 µL reaction volume containing 200 nM of each forward and reverse primer, and 50 ng of cDNA template. The thermal cycling conditions were as follows: initial denaturation at 95 °C for 3 min, followed by 40 cycles of denaturation at 95 °C for 10 s, annealing at 60 °C for 20 s, and extension at 72 °C for 30 s. Fluorescence data were acquired at the end of each extension step. The relative expression levels of *PDGFRα* or *KCNK3* were normalized to *GAPDH* and calculated using the 2^−ΔΔCt^ method to determine fold changes.

### 2.6. Statistical Analysis

Data are expressed as the mean ± S.D. Group differences were evaluated using one-way ANOVA followed by post hoc Bonferroni correction, performed with OriginPro 2020 software (OriginLab Corp., Northampton, MA, USA). Assumptions of normality and homogeneity of variance were verified using Shapiro–Wilk and Levene’s tests, respectively. All data points were included, and no outliers were excluded. A significance level of *p* < 0.01 was chosen to enhance robustness against Type I error in multiple comparisons, particularly given the apparent expression differences between tissues.

## 3. Results

### 3.1. Dynamic Expression Patterns of PDGFRα in Human and Mouse Tissues

**Figure 1:**
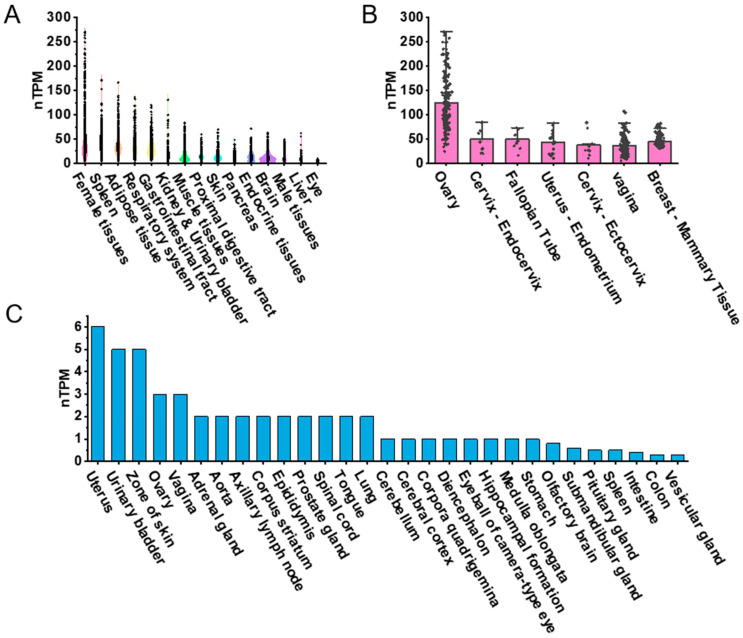
PDGFRα expression in human and mouse tissues based on public datasets. (**A**) Violin plot highlighting top 15 tissue types with highest PDGFRα expression levels in humans. (**B**) PDGFRα expression in seven female tissue types in humans. (**C**) Bar graph representing PDGFRα expression across 29 different tissue types in mice. Due to cross-platform and cross-species variability, comparisons were based on descriptive ranking and visual comparison of average expression values, rather than formal statistical tests.

To explore the expression patterns of PDGFRα across various human tissues, we visualized RNA-seq transcriptomic data from the GTEx BodyMAP using RNA-seq quantification tools available on the GTEx portal. Expression levels were quantified as transcripts per million (TPM) and normalized (nTPM) for tissue comparison. The dataset included approximately 1000 human samples representing 54 tissue types, such as female reproductive tissues, spleen, adipose tissue, respiratory system, gastrointestinal tract, kidney, urinary bladder, muscle tissues, proximal digestive tract, skin, pancreas, endocrine tissues, brain, male reproductive tissues, liver, and eyes (Figure 1A). We focused on female reproductive tissues, including the ovary, endocervix, fallopian tube, the endometrium of the uterus, ectocervix, vagina, and breast. The ovary exhibited the highest nTPM levels for PDGFRα expression (Figure 1B). While this ranking is based on visual inspection of expression values, no formal statistical test was conducted across tissues, given differences in sample size and inter-individual variance.

For mouse tissues, PDGFRα expression was obtained from the FANTOM5 CAGE dataset, and expression was also presented as nTPM. The tissues included uterus, urinary bladder, skin, ovary, vagina, adrenal gland, aorta, axillary lymph node, corpus striatum, epididymis, prostate gland, spinal cord, tongue, lung, cerebellum, cerebral cortex, corpora quadrigemina, diencephalon, eyeball (camera-type eye), hippocampus, medulla oblongata, stomach, olfactory brain, submandibular gland, spleen, intestine, colon, and vesicular gland (Figure 1C). PDGFRα expression was also observed in the mouse ovary, though comparisons between species were not statistically tested due to platform and normalization differences between GTEx RNA-seq and FANTOM5 CAGE datasets. Nonetheless, a descriptive trend suggested relatively higher PDGFRα expression in reproductive tissues in both species.

### 3.2. Expression Pattern of PDGFRα in PDGFRα^EGFP^ Mouse Ovary

**Figure 2:**
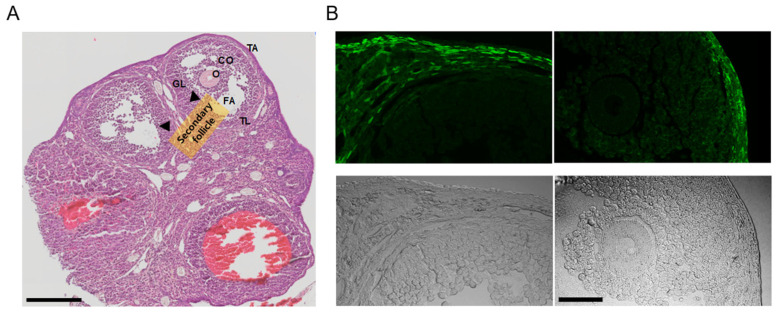
Analysis of ovarian tissue sections from PDGFRα^EGFP^ mice. (**A**) Hematoxylin and eosin (H&E)-stained images displaying the overall ovarian histology, with clearly identified secondary follicles (indicated by arrows). Granulosa cells (GLs) surround the oocyte (O), along with the cumulus oophorus (CO). The theca layers (TL) surrounding the follicles are visible. FA represents the follicular antrum, and TA represents the tunica albuginea. NC indicates the negative control condition without anti-KCNK3 antibody treatment. Scale bar, 200 μm. (**B**) Fluorescence and corresponding brightfield images of ovarian sections from PDGFRα^EGFP^ mice. The upper panels display EGFP fluorescence, and the lower panels show the matched brightfield views. The left panel displays a secondary follicle, while the right panel presents a Graafian follicle. The scale bar shown in the bottom right image applies to all panels in (**B**). Scale bar, 50 μm.

In the ovaries obtained from the PDGFRα^EGFP^ mouse model, H&E staining revealed typical ovarian histology, including the presence of secondary follicles. Secondary follicles are characterized by multiple layers of granulosa cells and the cumulus oophorus (CO) surrounding the oocyte. Small fluid-filled spaces, indicating early antral formation, were also observed within these follicles. The theca interna around the granulosa cell layer was clearly visible, with no detectable pathological changes (Figure 2A, n = 3).

The PDGFRα^EGFP^ mouse model is engineered to express EGFP in the nuclei of PDGFRα^+^ cells, allowing for the visualization of PDGFRα expression in various tissues. In this model, the EGFP signal indicates the presence of PDGFRα^+^ cells [26]. The fluorescence images (green) highlight the EGFP expression in the nuclei of PDGFRα^+^ cells. PDGFRα expression is predominantly observed in the thecal layer (TL) surrounding the granulosa cells in the stroma throughout the ovarian cortex. No detectable fluorescence signal was observed in granulosa cells or oocytes within the Graafian follicle under the imaging conditions (Figure 2B, n = 3).

### 3.3. Co-Localization of PDGFRα and KCNK3 in the Ovary

**Figure 3:**
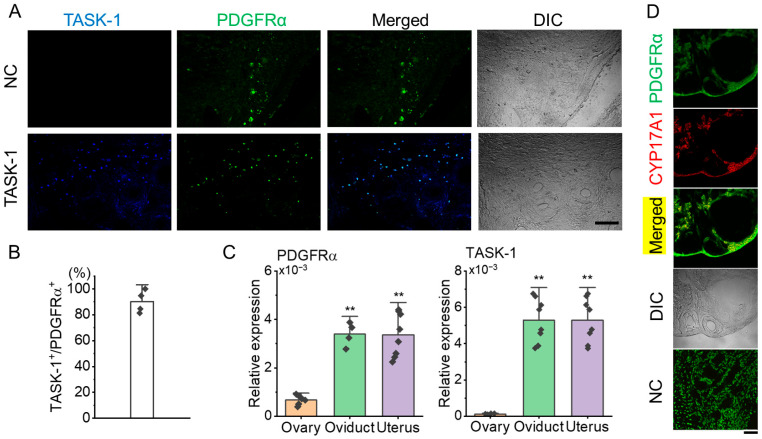
Co-localization of KCNK3 (TASK-1) and PDGFRα in ovarian tissue sections from PDGFRα^EGFP^ mice. (**A**) Co-localization of PDGFRα (green) and KCNK3 (blue) in ovarian tissues. Merged and differential interference contrast (DIC) images are shown to visualize cell structure. Scale bar, 50 μm. (**B**) Bar graph showing the percentage of KCNK3 co-localization with PDGFRα^+^ cells. (**C**) PDGFRα and KCNK3 transcript expression levels in the mouse ovary, oviduct, and uterus. Each bar represents the mean ± S.D. of biological replicates (sample size), with each sample measured in triplicate and averaged before statistical analysis. Sample size: ovary (n = 6), oviduct (n = 4), uterus (n = 8) for PDGFRα; ovary (n = 6), oviduct (n = 8), uterus (n = 8) for KCNK3 (TASK-1). ** *p* < 0.01 compared to the ovary. (**D**) Co-localization of PDGFRα and CYP17A1 in the thecal layer. PDGFRα (green) is expressed in ovarian stromal and thecal cells, while CYP17A1 (red) marks theca cells explicitly. Merged images indicate partial co-localization of PDGFRα and CYP17A1. DIC: Differential interference contrast image. NC: Negative control. Scale bar, 50 µm.

Immunohistochemical analysis showed that KCNK3 is expressed in PDGFRα^+^ cells within the ovarian stroma (Figure 3A). In the NC, where the anti-KCNK3 antibody was omitted, only PDGFRα expression was observed in the stroma without KCNK3 expression (Figure 3A). Quantitative analysis showed that 90.2 ± 8.7% of PDGFRα^+^ cells co-expressed KCNK3 (Figure 3B, n = 4 ovaries), indicating a high overlap.

Quantitative PCR (qPCR) was performed to assess PDGFRα mRNA levels in 6 ovaries, 4 oviducts, and 8 uteri. For KCNK3 expression analysis, qPCR was conducted using 6 ovaries, 8 oviducts, and 8 uteri. The qPCR results showed that PDGFRα expression in the ovary was lower than in both the oviduct and uterus. There was no significant difference in PDGFRα expression levels between the oviduct and uterus. Like PDGFRα, KCNK3 expression levels were lower in the ovary than in the oviduct and uterus (Figure 3C, *p* < 0.01). As shown in Figure 3D, PDGFRα was found to co-localize with 17α-hydroxylase (CYP17A1), a well-established marker of theca cells, in the thecal layer, supporting the presence of PDGFRα^+^ cells in this region. This finding confirms that a subset of theca cells expresses PDGFRα, suggesting its potential involvement in the steroidogenic function of these cells. The NC showed no detectable non-specific staining, confirming the specificity of the immunostaining.

## 4. Discussion

This study analyzes PDGFRα expression patterns across various human and mouse tissues using publicly available datasets. RNA expression data from the Human Protein Atlas reveal distinct tissue-specific expression profiles for PDGFRα, with a predominant presence in female reproductive tissues, particularly in the ovary. Similarly, CAGE data for mice show that PDGFRα is predominantly expressed in female tissues, particularly in the uterus and ovary. However, the expression levels of PDGFRα in human reproductive organs, such as the ovary and uterus, were markedly higher than those observed in corresponding mouse tissues. These interspecies differences may be attributed to several factors. First, hormonal regulation may differ significantly between humans and mice, particularly in the dynamics and levels of circulating estrogen, progesterone, and gonadotropins, which could influence PDGFRα expression. Second, differences in stromal cell composition and the density or subtype distribution of PDGFRα^+^ cells may contribute to the observed variability. Third, evolutionary divergence in gene regulatory elements or epigenetic modifications may lead to altered transcriptional activity of the Pdgfra gene across species. These considerations highlight the importance of cautious cross-species comparisons and suggest that while mouse models provide valuable insights, human-specific studies are necessary to fully elucidate PDGFRα’s role in reproductive physiology.

This study’s histological analysis of the *PDGFRα^EGFP^* mouse model confirmed the localization of PDGFRα expression in ovarian stromal cells, particularly within the thecal layer. This spatially restricted pattern suggests a selective role for PDGFRα in non-follicular stromal compartments, distinguishing its function from granulosa cells or oocytes. Although no detectable PDGFRα expression was observed in granulosa cells or oocytes, this does not preclude functional relevance. Expression may fluctuate during folliculogenesis or fall below detection thresholds, necessitating further stage-specific analyses to resolve transient or low-abundance expression.

Consistent with previous studies reporting PDGFRα enrichment in theca cells [34,35,36], our findings support the notion that PDGFRα^+^ cells represent a specialized subpopulation within the ovarian stroma. These cells may modulate follicular development via paracrine signaling. Given theca cells’ established role in androgen synthesis, subsequently aromatized to estrogens by granulosa cells [37], PDGFRα may contribute more directly to steroidogenic regulation. PDGF signaling has been implicated in mesenchymal cell recruitment, proliferation, and angiogenesis [9,38], suggesting that PDGFRα expression in theca cells could also reflect a role in progenitor cell differentiation into steroidogenic lineages, especially during early follicular development. This is supported by findings that PDGFRα^+^ ovarian progenitors can give rise to *CYP17A1^+^* and *CYP11A1^+^* cells [39]. Furthermore, PDGFRα^+^ stromal cells may facilitate vascular remodeling in the follicular microenvironment, enabling the delivery of cholesterol precursors and endocrine signals essential for follicular growth [14,40].

Our previous study identified PDGFRα expression in testicular Leydig cells, but not in germ or Sertoli cells [29]. Since Leydig and theca cells share interstitial localization and androgen-producing functions, PDGFRα may act as a conserved regulator of steroidogenic cell lineages in both male and female gonads. Notably, KCNK3, one of the most prominent K_2P_ potassium channels, co-localizes with PDGFRα in Leydig cells and, as we show here, also in ovarian stromal cells, including theca cells. This co-expression suggests a functional association, potentially linked to hormone biosynthesis or ionic homeostasis. In Leydig cells, KCNK3 blockade induces apoptosis, implying a role in cell survival [41]. Similarly, KCNK3 is highly expressed in jejunal PDGFRα^+^ cells and contributes to maintaining a hyperpolarized membrane potential [26,42]. Ovarian PDGFRα cells may share these electrophysiological properties, where KCNK3 could help establish a bioelectric environment conducive to steroidogenesis or signal transduction. Our findings highlight the potential of PDGFRα and KCNK3 as molecular markers and functional mediators in ovarian stromal physiology.

Quantitative PCR analysis revealed that PDGFRα and KCNK3 expression levels were lower in the ovary compared to the oviduct and uterus. This tissue-specific expression pattern suggests that PDGFRα^+^ stromal cells may fulfill broader roles in the uterus and oviduct, potentially contributing to tissue remodeling, cyclic regeneration, and structural support. In contrast, their function may be more specialized within the ovary, focused on steroidogenesis and follicular maturation. PDGFRα is likely expressed in fibroblasts and smooth muscle cells in the fallopian tubes and uterus, contributing to the higher expression levels observed in these tissues [43]. Moreover, the dynamic processes of endometrial regeneration and angiogenesis in the uterus, both highly dependent on PDGF signaling, may further account for elevated PDGFRα expression [44,45]. KCNK3, which co-expresses with PDGFRα, may serve as a functional marker for these stromal cells, regulating membrane potential, ion balance, and possibly cell proliferation in coordination with PDGFRα signaling.

The co-localization of PDGFRα and KCNK3 in ovarian stromal cells, particularly in the thecal layer, suggests a specialized subpopulation involved in hormonal and ionic regulation. As noted by Kinnear et al., the ovarian stroma is a functionally heterogeneous tissue comprising diverse fibroblast-like populations that contribute to structural integrity, angiogenesis, immune modulation, and paracrine signaling [36]. PDGFRα^+^/KCNK3^+^ cells may represent a hormonally responsive subset regulated by gonadotropins or sex steroids, playing dynamic roles during the estrous cycle. Supporting this, single-cell transcriptomic analyses have identified stromal clusters expressing PDGFRα alongside steroidogenic markers such as *CYP17A1* and *STAR* [46], consistent with our findings. Further molecular profiling is needed to delineate the precise function of these stromal subtypes in reproductive physiology.

In light of these findings, future studies should also explore the functional roles of PDGFRα^+^/KCNK3^+^ stromal cells beyond spatial localization. While the current research is primarily descriptive, we acknowledge the lack of functional validation as a key limitation. Approaches such as gene silencing of PDGFRα or KCNK3, pharmacological modulation of PDGFRα or KCNK3 activity, or hormone-response assays (e.g., under LH or FSH stimulation) will be essential to determine the causal involvement of these cells in steroidogenesis, folliculogenesis, and stromal remodeling. Investigating whether KCNK3 influences membrane potential or modulates PDGF signaling under endocrine regulation will help clarify whether the observed co-expression reflects a physiologically significant regulatory mechanism.

Although this study offers novel insights into the spatial distribution and co-localization of PDGFRα and KCNK3 in the mouse ovary, several limitations should be acknowledged. Nuclear counterstaining (e.g., DAPI) was intentionally omitted to avoid interference with the nuclear-localized EGFP in the PDGFRα^EGFP^ mouse model, potentially limiting precise nuclear demarcation. Conventional positive controls using overexpressed proteins were not included due to the absence of a CYP17A1 construct and the use of conjugated primary antibodies on KCNK3 to minimize non-specific signals. Nevertheless, appropriate negative controls and signal distributions consistent with known expression patterns support staining specificity. Furthermore, expression was not assessed systematically across distinct follicular stages due to imaging and sectioning constraints. Fluorescence quantification was based on manual counts of signals above background in defined regions, without automated or blinded analysis. Future studies should incorporate threshold-based or machine learning-guided image analysis to enhance objectivity and reproducibility.

Despite these limitations, our findings provide a valuable foundation for future mechanistic studies on the role of stromal ion channels and PDGF signaling in ovarian physiology and reproductive disorders.

## 5. Conclusions

In conclusion, this study highlights the distinct expression patterns of PDGFRα across human and mouse tissues, with the ovary showing high expression in both species, although it is more pronounced in humans. In the PDGFRα^EGFP^ mouse model, PDGFRα was localized primarily to thecal cells in the ovarian stroma, suggesting a role in steroidogenesis and follicular development. Additionally, significant co-localization of PDGFRα and KCNK3 in the ovary points to potential functional interactions in regulating reproductive physiology. These findings suggest that PDGFRα and KCNK3 may play essential roles in ovarian function and may be involved in broader reproductive processes.

## Figures and Tables

**Table 1:** Primer sequences used for qPCR.

Gene Name	Species	GenBank Accession Numbers	Primer Sequences (5′–3′)	Expected Size (bp)
*Pdgfra*	Mouse	BC053036.1	F: TGCGGGTGGACTCTGATAATGC R: GTGGAACTACTGGAACCTGTCTCG	235
*KCNK3*	Mouse	NM_001083316.2	F: TCCTTCTACTTCGCCATCACC R: AGGCTCTGGAACATGACTAGTGT	137
*Gapdh*	Mouse	GU214026.1	F: AC CAGAAGACTGTGGATGG R: CACATTGGGGGTAGGAACAC	171

## Data Availability

Publicly available transcriptomic datasets used in this study were obtained from the Genotype-Tissue Expression (GTEx) project (https://gtexportal.org/home/, accessed on 24 July 2024) and the FANTOM5 Cap Analysis of Gene Expression (CAGE) dataset (https://fantom.gsc.riken.jp/5/, accessed on 24 July 2024). The accession number for the GTEx data is phs000424.v8.p2. Additional experimental data generated during this study (e.g., qPCR, immunostaining) are available from the corresponding author upon reasonable request.

## Abbreviations

The following abbreviations are used in this manuscript:

KCNK3	potassium two-pore domain channel subfamily K member 3
PDGFRα	platelet-derived growth factor receptor alpha
TASK-1	tandem of P domains in a weak inward rectifying K^+^ channel-related acid-sensitive K^+^-1
